# BDE-47 Induces Mitochondrial Dysfunction and Endoplasmic Reticulum Stress to Inhibit Early Porcine Embryonic Development

**DOI:** 10.3390/ani13142291

**Published:** 2023-07-13

**Authors:** Rong-Ping Liu, Sheng-Yan He, Jing Wang, Xin-Qin Wang, Zhe-Long Jin, Hao Guo, Chao-Rui Wang, Yong-Nan Xu, Nam-Hyung Kim

**Affiliations:** 1 Guangdong Provincial Key Laboratory of Large Animal Models for Biomedicine, Wuyi University, Jiangmen 529020, China; 2 College of Agriculture, Yanbian University, Yanji 133002, China

**Keywords:** BDE-47, embryonic development, antioxidant, endoplasmic reticulum, mitochondrial

## Abstract

**Simple Summary:**

The 2,2′4,4′-tetrabromodiphenyl ether (BDE-47) is a common flame retardant that can be widely distributed in the environment and organisms but has been shown to induce toxicity to various organisms and organ systems. We found that exposure to BDE-47 induced the early embryonic development of in vitro porcine culture through oxidative stress and autophagy induced by mitochondrial dysfunction and endoplasmic reticulum stress.

**Abstract:**

Widely used as a flame retardant, 2,2′4,4′-tetrabromodiphenyl ether (BDE-47) is a persistent environmental pollutant with toxicological effects, including hepatotoxicity, neurotoxicity, reproductive toxicity, and endocrine disruption. To investigate the toxicological effects of BDE-47 on early porcine embryogenesis in vitro, cultured porcine embryos were exposed to BDE-47 during early development. Exposure to 100 μM BDE-47 decreased the blastocyst rate and mRNA level of pluripotency genes but increased the level of LC3 and the expression of autophagy-related genes. After BDE-47 exposure, porcine embryos’ antioxidant capability decreased; ROS levels increased, while glutathione (GSH) levels and the expression of antioxidant-related genes decreased. In addition, BDE-47 exposure reduced mitochondrial abundance and mitochondrial membrane potential levels, downregulated mitochondrial biogenesis-associated genes, decreased endoplasmic reticulum (ER) abundance, increased the levels of GRP78, a marker of ER stress (ERS), and upregulated the expression of ERS-related genes. However, ER damage and low embryo quality induced by BDE-47 exposure were reversed with the ERS inhibitor, the 4-phenylbutyric acid. In conclusion, BDE-47 inhibits the development of early porcine embryos in vitro by inducing mitochondrial dysfunction and ERS. This study sheds light on the mechanisms of BDE-47-induced embryonic toxicity.

## 1. Introduction

Polybrominated biphenyl ethers (PBDEs) are organic and persistent environmental pollutants of wide concern because of their accumulation and toxicity in organisms [1]. The 2,2′4,4′-tetrabromodiphenyl ether (BDE-47) is a PBDE homolog widely distributed in the environment, the human placenta, body fluids, and the umbilical cord [2,3,4]. BDE-47 is widely used as a flame retardant in textiles, building materials, and plastics. However, it exhibits widespread biological toxicity, including reproductive toxicity, liver toxicity, neurotoxicity, and endocrine interference [5,6,7]. BDE-47 induces ovarian and uterine damage and disrupts the oxidative balance and mitochondrial function in mouse oocytes [8]. BDE-47 also induces hepatotoxicity and neurodevelopmental toxicity in zebrafish larvae [9] and damages hearing by inducing cochlear hair cell necrosis [10]. Therefore, exposure to BDE-47 is hazardous to cellular homeostasis.

Mitochondria are the main sites of cellular energy production [11,12] and play a key role in the regulation of cell growth, information transfer, and apoptosis [13,14]. Mitochondria are also needed for oxidative phosphorylation to provide energy during gametogenesis, fertilization, and early embryonic development [15]. Mitochondrial homeostasis is essential for mammalian reproductive processes [11,16], and mitochondrial dysfunction leads to oxidative stress [13]. The production of reactive oxygen species (ROS) exceeds the ability of the antioxidant system to clear them in a phenomenon known as oxidative stress. Excess ROS can cause DNA damage, mitochondrial damage, apoptosis, and autophagy [17,18], and oxidative stress can cause oocyte aging and inhibit embryonic development [19].

Mitochondria and the endoplasmic reticulum (ER) are adjacent in cells. The ER is the site of protein synthesis, processing, and transport and a key organelle involved in the synthesis and metabolism of lipids and carbohydrates [20]. One of the crucial roles of the ER is to provide proper protein synthesis and processing during oocyte maturation, fertilization, and early embryonic development [21]. However, when the unfolded or misfolded proteins in the ER lumen exceed the folding capacity of the ER as a result of increased protein synthesis or environmental influences (e.g., hypoxia, nutrient deficiency, cellular damage), the cells activate a protective defense mechanism called ER stress (ERS). ERS triggers several processes, such as the unfolded protein response, ER overload response, and apoptotic autophagy, to re-establish ER homeostasis and functionality since severe or persistent ERS leads to cell dysfunction and, eventually, cell death [22,23]. Research shows that ERS induces apoptosis of ovarian granulosa cells [24], and prolonged ERS can inhibit embryonic development, thus impairing the reproductive process [21,25].

Previous studies have shown that BDE-47 exposure affects the in vivo maturation of mouse oocytes, but the effect of BDE-47 on early embryonic development in mammals is not yet known. As mentioned above, early embryo development relies heavily on mitochondrial activity for maintaining the cellular redox balance and ER environmental balance. Therefore, in this investigation, we focused on how BDE-47 affected early porcine embryonic homeostasis of mitochondria and ER. After supplying BDE-47 to early porcine embryos, we evaluated the blastocyst rate, autophagy levels, the changes in antioxidant capacity, the homeostasis of ER and mitochondrial, and the expression of related genes in early porcine embryos exposed to BDE-47.

## 2. Materials and Methods

All chemicals and reagents, unless otherwise specified, were bought from Sigma-Aldrich (St. Louis, MO, USA).

### 2.1. Oocyte Collection and In Vitro Maturation (IVM)

Porcine ovaries were transported from neighboring slaughterhouses to laboratories in thermos flasks filled with 37 °C sterile saline. The porcine ovaries used in the experiment were from slaughtered sows that had not been sacrificed for research. The ovaries were cleaned three times with sterile saline. Using a sterile syringe and an 18-gauge needle, the follicular fluid that contained cumulus-oocyte complexes (COCs) from ovarian follicles with a diameter of 3–8 mm was collected. COCs were cleaned five times with Tyrode’s lactate 4-(2-hydroxyethyl)-1-piperazineëthanesulfonic acid (HEPES) and then transferred to a clean IVM medium (M199 medium with 0.022 mg/mL sodium pyruvate, 10% porcine follicular fluid, 0.09 mg/mL L-cysteine, 1% penicillin-streptomycin, 10 IU/mL follicle-stimulating hormone, 20 ng/mL epidermal growth factor, and 10 IU/mL luteinizing hormone). Each well of a four-well culture plate was filled with 500 μL of IVM and roughly 100 oocytes, and each well was then filled with 500 μL of mineral oil to completely cover the IVM and oocytes. The oocytes spent 44 h at 38.5 °C (5% CO_2_, 100% humidity) for in vitro maturation. During this period, the IVM did not need to be replaced.

### 2.2. Parthenogenetic Activation and In Vitro Embryo Culture

In a solution containing 0.2% hyaluronidase, COCs were gently removed after IVM by pipetting the solution roughly 30 times. Denuded oocytes were subject to parthenogenetic activation with 300 mM mannitol, including 0.05 mM CaCl_2_, 0.5 mM HEPES, 0.1 mg/mL polyvinyl alcohol (PVA), and 0.1 mM MgSO_4_, with a direct current pulse at 120 V stimulation twice, each time for 60 μs, separated by a 0.1 s break. After activation, embryos were cultivated in an in vitro culture (IVC) medium (bicarbonate-buffered PZM-5, containing 4 μg/μL bovine serum albumin [BSA]), containing 15.64 mM cytochalasin B for 3 h, moved to a clean IVC medium after being five times cleaned with the IVC medium. In 4-well plates, about 60 μL of the IVC medium totally covered by 600 μL of mineral oil was used to culture roughly 40 embryos per well at 38.5 °C for 7 days (5% CO_2_, 100% humidity). The culture media contained different concentrations of BDE-47 (0, 50, 100, and 200 μM), while the control medium had no BDE-47. The blastocyst rate was detected on day 7 after BDE-47 treatment.

Dimethyl sulfoxide (DMSO) was used to dissolve BDE-47 powder (714157, TMstandard, Beijing, China) and the ERS inhibitor, 4-phenylbutyrate (PBA powder, HY-A0281, MedChemExpress, Guangzhou, China). These stocks were then diluted to the final concentration by the IVC medium for the experiments. The DMSO was present in less than 0.025% of the IVC medium. In order to reduce any influence of DMSO, the IVC medium with 0.025% DMSO was used as the control group.

### 2.3. Assessment of Mitochondrial Abundance

The embryos were cultured in IVC medium containing 100 μM BDE-47 for 3 days; the embryos developed to the four-cell stage; then, the embryos were collected, and after five cleanings in phosphate-buffered saline (PBS)-PVA, the abundance of mitochondria in embryos was measured. The embryos were then subjected to a 1 h treatment in PBS-PVA, adding 200 nM MitoTracker Red CMXRos (Beyotime, Shanghai, China) at 37 °C in the dark. After five cleanings in PBS-PVA, the embryos were placed in a new PBS-PVA. A fluorescence microscope (Ti2, Nikon, Tokyo, Japan) was used to image, and ImageJ (NIH, Bethesda, MD, USA) software was used to analyze the red fluorescence intensity.

### 2.4. Assessment of Mitochondrial Membrane Potential (MMP, ΔΨm) 

The embryos were cultured in an IVC medium containing 100 μM BDE-47 for 3 days; then, the four-cell stage embryos were gathered, and the MMP in embryos was quantified after being washed five times in PBS-PVA. The embryos were then co-incubated in 10 µM 5,5′,6,6′-tetrachloro-1,1′,3,3′-tetraethylbenzimidazolylcarbocyanine iodide (JC-1; Invitrogen, Rochester, NY, USA) diluted with PBS-PVA for 1 h in a 37 °C dark environment. The embryos were washed five times in PBS-PVA and then placed in new PBS-PVA. The images were taken using the fluorescence microscope, and the intensity of red and green fluorescence was analyzed using ImageJ software (version 8.0.2; NIH, Bethesda, MD, USA). The levels of embryonic MMP were determined using the ratio of red fluorescence intensity (j-aggregates) to green fluorescence intensity (j-monomers). 

### 2.5. Assessment of Intracellular ROS and Glutathione (GSH) Levels

The embryos were cultured in an IVC medium containing 100 μM BDE-47 for 3 days, then the four-cell stage embryos were collected and then washed five times in PBS-PVA to detect the amounts of ROS and GSH. The embryos were then co-incubated in 10 µM 2′,7′-dichlorodihydrofluorescein diacetate (DCFH, Beyotime) diluted with PBS-PVA for 45 min in a 37 °C dark environment and then washed five times in PBS-PVA to analyze ROS levels. The embryos were co-incubated for 30 min in a 37 °C dark environment in PBS-PVA with 10 µM 4-chloromethyl-6,8-difluoro-7-hydroxycoumarin (CMF2HC, Invitrogen, Carlsbad, CA, USA) and then rinsed five times with PBS-PVA to detect GSH levels. The washed embryos were transferred to fresh PBS-PVA. A fluorescence microscope was used to image, and the intensity of blue and green fluorescence was analyzed using ImageJ software.

### 2.6. Assessment of ER Abundance

To measure ER abundance, embryos were co-cultured with 100 μM BDE-47 for 3 days; then, the four-cell stage embryos were gathered and washed five times in PBS-PVA. The embryos were then placed in PBS-PVA containing 2.5 µM ER-Tracker Red (Beyotime) and incubated at 37 °C for 1 h in the dark. The embryos were washed five times with PBS-PVA and then placed in fresh PBS-PVA. A fluorescence microscope was used to image, and ImageJ software was used to analyze the red fluorescence intensity.

### 2.7. Immunofluorescence Staining

The embryos were cultured in IVC medium containing 100 μM BDE-47 for 7 days; blastocysts were obtained, rinsed five times using PBS-PVA, and then maintained in PBS-PVA with 3.7% paraformaldehyde for 30 min at room temperature. Blastocysts were permeabilized using 0.3% Triton X-100 for 30 min at room temperature and then placed in PBS-PVA with 3% BSA to block at 37 °C for 1 h. The blastocysts were incubated with a rabbit anti-LC3B antibody (#ab48394, Abcam, Cambridge, UK, 1:200) and rabbit anti-GRP78 antibody (#3177, Cell Signaling, Technology, Boston, MA, USA, 1:300) at 4 °C overnight. After five washes with PBS-PVA, blastocysts were treated with a goat anti-rabbit IgG antibody (#ab150077, Abcam, 1:1000) for 1 h in a 37 °C dark environment. To label the nuclei, the blastocysts were treated for 10 min at 37 °C with 10 μg/mL of Hoechst 33342. Finally, the blastocysts were glued to glass slides using anti-fluorescence attenuation sealant after five rinsings in PBS-PVA. The images were taken using the fluorescence microscope, and the intensity of green fluorescence was analyzed using ImageJ software. The relative fluorescence intensity of LC3B was used to assess the level of autophagy, while the relative fluorescence of GRP78 was used to assess the level of ERS.

### 2.8. Reverse-Transcription—Quantitative Polymerase Chain Reaction (RT-qPCR) Analysis

The embryos were cultured in IVC medium containing 100 μM BDE-47 for 7 days; total RNA was isolated from 35 blastocysts per pool using the Dynabeads mRNA DIRECT Purification Kit (Invitrogen, Carlsbad, CA, USA). RNA was reverse-transcribed into cDNA using the SuperScript III First Strand cDNA Synthesis Kit (Invitrogen). The KAPA SYBR FAST Universal qPCR Kit (Kapa Biosystems, Boston, MA, USA) was used for gene expression analysis. The total sample volume is 20 μL, including 1 μL for each gene-specific primer (10 pmol), 10 μL for KAPA SYBR FAST qPCR Master Mix (2×) Universal, 7 μL for deionized water, and 1 μL for the cDNA sample. The following were the conditions for the quantitative PCR reaction: polymerase was activated at 95 °C for 180 s; 40 cycles of denaturation for 3 s at 95 °C, annealing for 30 s at 60 °C, and elongation for 20 s at 72 °C and final extension at 72 °C for 5 min. *GAPDH* transcription levels were used for standardization. Gene expression was quantified using the 2^−ΔΔCt^ method. Each cDNA sample was analyzed three times in triplicate. Three triplicate analyses were performed on each cDNA sample. Table 1 lists the primers used for the RT-qPCR.

### 2.9. Statistical Analysis

The mean ± standard deviation (SD) of the data is presented. The measured quantities were expressed as fold-control after normalizing to the control values unless otherwise mentioned. The figure legends provide information on the number of embryos (N) utilized in each experiment, as well as the number of times it was carried out. The statistical analyses were performed by SPSS software (version 26.0; IBM, Chicago, IL, USA). A Student’s *t*-test was used to analyze the differences between the groups. Three or more groups of data were analyzed by one-way analysis of variance (Tukey–Kramer test). 

For the calculation of fluorescence intensity values, we obtained fluorescence intensity for each embryo using ImageJ and then normalized the fluorescence intensity to 1 in the control group embryos. Then, the relative fluorescence intensities of the control group and the experimental group were calculated. First, the average values of fluorescence intensity in the control embryos were calculated, and the ratio of the fluorescence intensity value of each embryo in the control group and the experimental group to the average value of the fluorescence intensity of the control group represents the relative fluorescence intensity value of each embryo.

To assess IC_50_, we used Prism software to transform concentrations, then Nonlinear regression of them as the *X*-axis and the inhibition rate as the *Y*-axis; then, we obtained a concentration of BDE-47 with an inhibition rate of 50%.

## 3. Results

### 3.1. Effect of BDE-47 on Early Embryonic Development In Vitro

**Figure 1:**
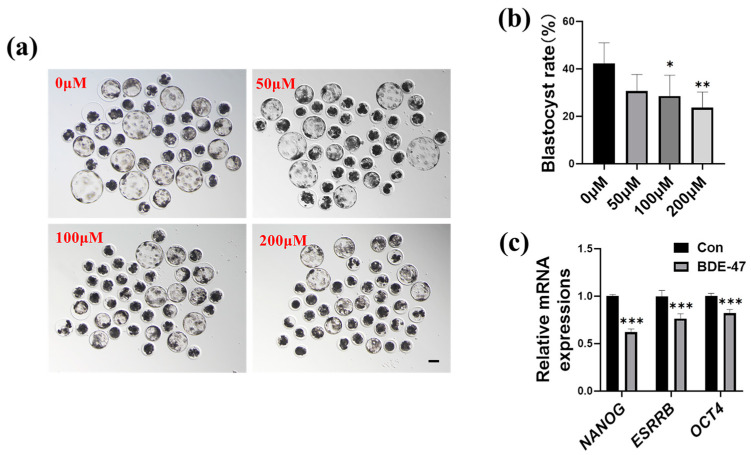
Effects of varying BDE-47 concentrations (0, 50, 100, and 200 μM) on the in vitro formation of porcine blastocysts. (**a**) Typical images of embryos after being exposed to 0, 50, 100, and 200 μM BDE-47 for 7 days. Scale bar, 100 μm. (**b**) Rates of blastocyst formation in embryos co-incubated with various BDE-47 concentrations. The number of embryos (*N*) used in this experiment was 168, 167, 164, and 165 for the 0, 50, 100, and 200 μM BDE-47 groups, respectively (4 replicates). (**c**) Statistics on the embryos’ relative mRNA amounts of pluripotency-related genes, such as *NANOG*, *ESRRB*, and *OCT4* in embryos were examined using RT-qPCR (3 replicates); The concentration of BDE-47 was 100 μM. The mean ± SD is presented for the data. *, *p* < 0.05; **, *p* < 0.01; ***, *p* < 0.001.

First, we studied the effect of BDE-47 on blastocyst formation. Early porcine embryos’ in vitro development was hampered after exposure to BDE-47 (Figure 1a–c). In embryos treated with 0, 50, 100, and 200 μM BDE-47, the rates of blastocyst formation on Day 7 were 42.28 ± 8.66%, 30.64 ± 7.04%, 28.57 ± 8.06%, and 23.68 ± 6.59%, respectively. The blastocyst rate decreased gradually with the increase in BDE-47 concentration. At the concentration of 100 μM, the blastocyst rate decreased significantly. Thus, the subsequent studies employed this concentration (100 μM). In addition to decreasing the blastocyst rate, exposure to BDE-47 significantly reduced the mRNA levels of pluripotency-related genes, such as *NANOG*, *ESRRB*, and *OCT4* in embryos (0.62 ± 0.03, 0.76 ± 0.05, and 0.82 ± 0.04, respectively; Figure 1c). Therefore, BDE-47 inhibits blastocyst formation and embryo pluripotency.

### 3.2. BDE-47 Promotes Autophagy and Reduces Total Cell Number in Early Porcine Embryos

**Figure 2:**
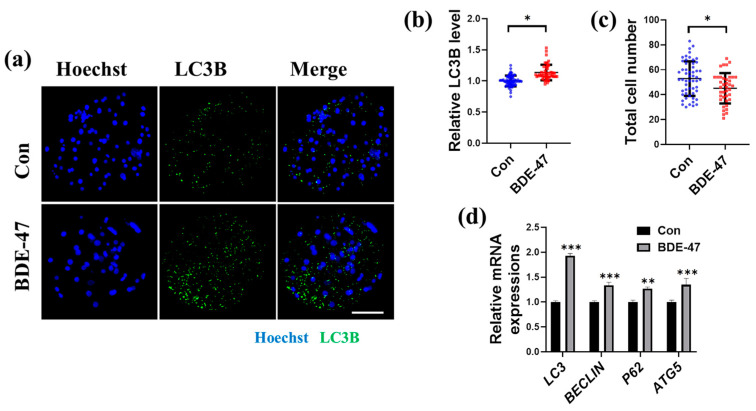
Effect of BDE-47 on early embryonic autophagy. (**a**) A typical image of embryos stained with Hoechst 33342 (blue) and LC3B (green). Scale bar, 100 μm. (**b**) Statistics on the relative LC3B fluorescence intensity levels in embryos treated with (Number of embryos, *N* = 64) or without (Number of embryos, *N* = 47) BDE-47 (4 replicates); Blue dots represent control embryos, red dots represent BDE-47-treated embryos. (**c**) Total cell number of embryos in control (Number of embryos, *N* = 54) or BDE-47-treated (Number of embryos, *N* = 40) groups (3 replicates); Blue dots represent control embryos, red dots represent BDE-47-treated embryos. (**d**) Statistics on the relative mRNA amounts of autophagy-related genes, such as *LC3*, *BECLIN*, *p62*, and *ATG5*, in embryos were examined using RT-qPCR (3 replicates). The concentration of BDE-47 was 100 μM. The mean ± SD is presented for the data. *, *p* < 0.05; **, *p* < 0.01; ***, *p* < 0.001.

BDE-47 has been shown to impair embryonic development, and previous studies reported that BDE-47 could induce placental toxicity and developmental neurotoxicity by increasing autophagy levels in mice and rats [26,27,28]. Therefore, we also studied the effect of BDE-47 on early porcine embryos’ autophagy. After 7 days of BDE-47 treatment, the expression of genes associated with autophagy and the amounts of autophagy marker LC3B in embryos were analyzed. The amount of the autophagy marker LC3B and the expression of genes associated with autophagy in embryos were assessed after 7 days of BDE-47 treatment. LC3B’s relative fluorescence intensity was dramatically increased to 1.14 ± 0.12 after being exposed to BDE-47 (Figure 2a,b). In addition, the total cell number in embryos decreased after BDE-47 treatment (Figure 2a,c). Additionally, treatment with BDE-47 increased the mRNA amounts of autophagy-related genes, such as *LC3*, *BECLIN*, *p62*, and *ATG5* (1.93 ± 0.04, 1.34 ± 0.06, 1.27 ± 0.03, and 1.35 ± 0.13, respectively; Figure 2d). These results show that exposure of early embryos to BDE-47 increases autophagy levels and reduces total cell number in vitro.

### 3.3. BDE-47 Disturbs the Oxidative Balance in Early Porcine Embryos

**Figure 3:**
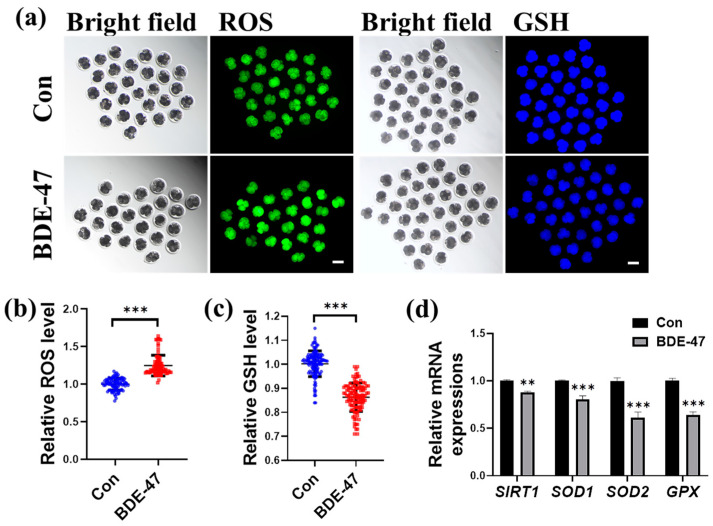
Effect of BDE-47 on early porcine embryonic oxidative balance. (**a**) A typical image of ROS (green) and GSH (blue) staining. Scale bar, 100 μm. (**b**) Statistics on the relative ROS amounts in embryos incubated with (Number of embryos, *N* = 83) or without (Number of embryos, *N* = 80) BDE-47 (3 replicates); Blue dots represent control embryos, red dots represent BDE-47-treated embryos. (**c**) Statistics on the relative amounts of GSH in embryos incubated with (Number of embryos, *N* = 122) or without (Number of embryos, *N* = 114) BDE-47 (3 replicates); Blue dots represent control embryos, red dots represent BDE-47-treated embryos. (**d**) Statistics on the relative mRNA amounts of antioxidant-related genes, such as *SIRT1*, *SOD1*, *SOD2*, and *GPX* in embryos, were examined using RT-qPCR (3 replicates). The concentration of BDE-47 was 100 μM. The mean ± SD is presented for the data. **, *p* < 0.01; ***, *p* < 0.001.

Oxidative stress is associated with defects in embryonic development. Therefore, the effect of BDE-47 on the oxidative balance of early embryos was studied. We analyzed the levels of ROS and the endogenous antioxidant, GSH, in four-cell stage embryos. Compared to the control group, the relative levels of ROS in the BDE-47-treated group were significantly higher (1.24 ± 0.14; Figure 3a,b), whereas the relative levels of GSH were significantly lower (0.86 ± 0.06; Figure 3a,c). In embryos treated with BDE-47, the antioxidant-associated genes, such as *SIRT1*, *SOD1*, *SOD2*, and *GPX,* were downregulated (0.88 ± 0.01, 0.81 ± 0.04, 0.61 ± 0.06, and 0.64 ± 0.03, respectively; Figure 3d). Our results suggest that BDE-47 treatment induces oxidative stress.

### 3.4. BDE-47 Impairs the Mitochondrial in Early Porcine Embryos

**Figure 4:**
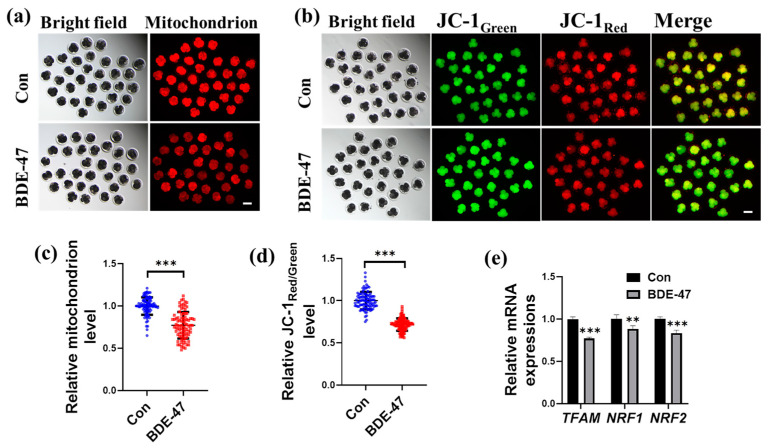
Effect of BDE-47 on mitochondrial in early embryos. (**a**) A typical image of MitoTracker Red staining. Scale bar, 100 μm. (**b**) A typical image of JC-1 staining. Scale bar, 100 μm. (**c**) Statistics on the relative amounts of mitochondria in embryos incubated with (Number of embryos, *N* = 88) or without (Number of embryos, *N* = 87) BDE-47 (3 replicates); Blue dots represent control embryos, red dots represent BDE-47-treated embryos. (**d**) Statistics on the relative intensity of JC-1 red/green fluorescence in embryos incubated with (Number of embryos, *N* = 93) or without (Number of embryos, *N* = 92) BDE-47 (3 replicates); Blue dots represent control embryos, red dots represent BDE-47-treated embryos. (**e**) Statistics on the relative mRNA amounts of the genes *TFAM*, *NRF1*, and *NRF2,* which are associated with mitochondrial biogenesis in embryos, were examined using RT-qPCR (3 replicates). The concentration of BDE-47 was 100 μM. The mean ± SD is presented for the data. **, *p* < 0.01; ***, *p* < 0.001.

Oxidative stress can lead to mitochondrial dysfunction. Therefore, the effect of BDE-47 on mitochondrial function, mitochondrial abundance, mitochondrial membrane potential (MMP) amounts, and the mRNA amounts of related genes in porcine embryos were examined. After BDE-47 treatment, embryos at the four-cell stage had considerably lower levels of fluorescence intensity in mitochondria than that in the control group (0.77 ± 0.16; Figure 4a,c). Compared with the control group, the intensity of JC-1 red/green fluorescence decreased 0.72 ± 0.07-fold after the embryos were co-incubated with BDE-47 (Figure 4b,d), and BDE-47 significantly reduced the MMP. Furthermore, the mRNA amounts of the genes *TFAM*, *NRF1*, and *NRF2* that are involved in mitochondrial biogenesis were also reduced in embryos incubated with BDE-47 (0.77 ± 0.02, 0.88 ± 0.04, and 0.83 ± 0.04, respectively; Figure 4e). These results suggest that BDE-47 treatment impairs mitochondrial homeostasis.

### 3.5. BDE-47 Impairs ER Function of Early Porcine Embryos

**Figure 5:**
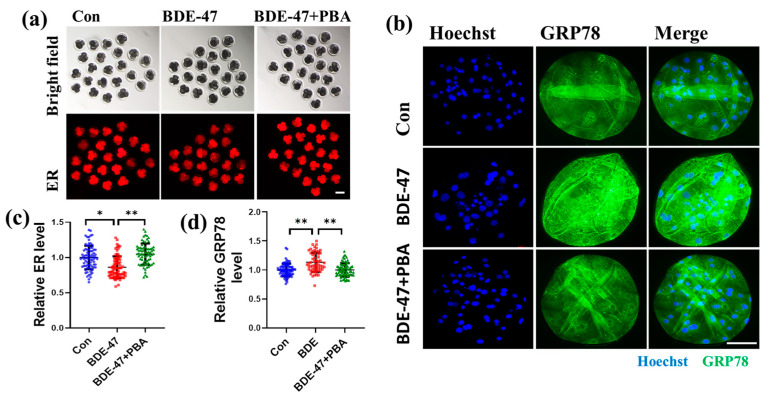
Effect of BDE-47 on early embryonic ER function in porcine. (**a**) A typical image of embryos stained with ER-Tracker Red. Scale bar, 100 μm. (**b**) A typical image of embryos stained with Hoechst 33342 (blue) and GRP78 (green). Scale bar, 100 μm. (**c**) Statistics on the relative amounts of ER in embryos of the control (Number of embryos, *N* = 79), BDE-47-treated (Number of embryos, *N* = 77), and BDE-47+PBA-treated (Number of embryos, *N* = 72) groups (3 replicates); Blue dots represent control embryos, red dots represent BDE-47-treated embryos, green dots represent BDE-47+PBA-treated embryos. (**d**) Statistics on the relative intensity of GRP78 fluorescence in embryos of the control (Number of embryos, *N* = 77), BDE-47-treated (Number of embryos, *N* = 57), BDE-47+PBA-treated (Number of embryos, *N* = 71) groups (3 replicates); Blue dots represent control embryos, red dots represent BDE-47-treated embryos, green dots represent BDE-47+PBA-treated embryos. The concentration of BDE-47 was 100 μM. The concentration of PBA was 75 μM. The mean ± SD is presented for the data. *, *p* < 0.05; **, *p* < 0.01.

**Figure 6:**
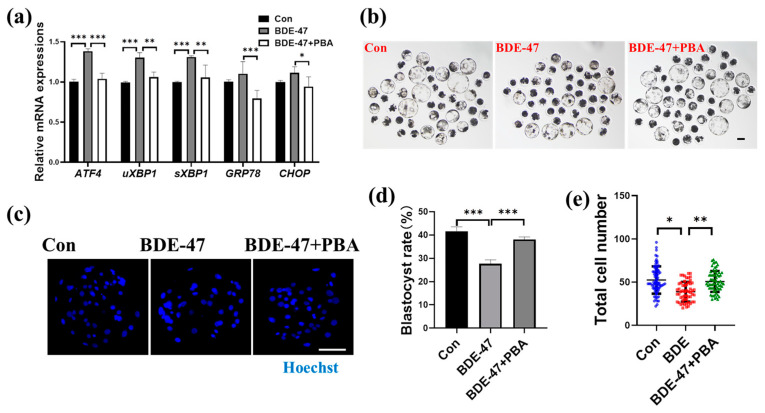
Effect of BDE-47 on ER homeostasis and early embryonic development. (**a**) Relative mRNA amounts of ERS-related genes *ATF4*, *uXBP1*, *sXBP1*, *GRP78*, and *CHOP* in embryos were examined using RT-qPCR (3 replicates). (**b**) A typical image of blastocyst formation in control, BDE-47-treated, and BDE-47+PBA-treated embryos. Scale bar, 100 μm. (**c**) Cell labeling images of embryos by Hoechst 33342 (blue). Scale bar, 100 μm. (**d**) Blastocyst formation rates in embryos of the control (Number of embryos, *N* = 195), BDE-47-treated (Number of embryos, *N* = 196), BDE-47+PBA-treated (Number of embryos, *N* = 197) groups (4 replicates). (**e**) Total cell number of embryos in control (Number of embryos, *N* = 74), BDE-47-treated (Number of embryos, *N* = 55), and BDE-47+PBA-treated (Number of embryos, *N* = 68) groups (3 replicates); Blue dots represent control embryos, red dots represent BDE-47-treated embryos, green dots represent BDE-47+PBA-treated embryos. The concentration of BDE-47 was 100 μM. The concentration of PBA was 75 μM. The mean ± SD is presented for the data. *, *p* < 0.05; **, *p* < 0.01; ***, *p* < 0.001.

The ER and mitochondria are structurally and functionally related. Therefore, the effect of BDE-47 on early porcine embryonic ER function was also studied. The ER abundance and the expression of the ERS marker GRP78 were assessed. Compared to the control group, the ER abundance in the BDE-47-treated group was considerably reduced (0.87 ± 0.17; Figure 5a,c), but this decrease was restored with the addition of the ERS inhibitor 4-phenylbutyrate (PBA) (Figure 5a,c). Contrasted with the control group, the BDE-47-treated group’s level of the ERS marker GRP78 was dramatically higher (1.13 ± 0.17; Figure 5b,d) but was restored after the addition of PBA (Figure 5b,d). Moreover, BDE-47 upregulated the expression levels of ERS-related genes in embryos (*ATF4*: 1.38 ± 0.03, *uXBP1*: 1.30 ± 0.06, *sXBP1*: 1.31 ± 0.02, *GRP78*: 1.10 ± 0.15, and *CHOP*: 1.11 ± 0.08; Figure 6a), and these changes were restored after the addition of PBA (Figure 6a). Although there was no significant difference in mRNA upregulation between *GRP78* and *CHOP*, there was a trend of mRNA upregulation, whereas the mRNA expression was significantly downregulated after the addition of PBA compared with the BDE-47 exposure group. After BDE-47 treatment, the blastocyst rate and total cell number of embryos decreased; however, inhibiting ERS with PBA reversed the decrease in blastocyst rate and total cell number in the BDE-treated group (Figure 6b–e). These results show that BDE-47 treatment impairs ER function and embryo development, but PBA can reverse this impairment.

## 4. Discussion

BDE-47 is widely used in various products because of its high flame-retardant efficiency, high stability, and low cost [29]. However, BDE-47 is difficult to decompose and easily accumulates in living organisms, including humans, and the local environment, causing environmental pollution and affecting human health [30,31].

In this study, we identified the deleterious effects of BDE-47 during early in vitro development in porcine embryos. BDE-47-treated porcine embryos showed lower developmental competence, decreased blastocyst rate and total cell number, and increased levels of embryo autophagy. In addition, BDE-47 treatment increased embryonic oxidative damage, mitochondrial damage, and ER dysfunction. However, the ERS inhibitor, PBA, alleviated the ER damage induced by BDE-47. Collectively, these data indicate that BDE-47 is toxic to early embryogenesis. BDE-47 is durable and bioaccumulates, with a half-life of up to 3 years [32]. In this experiment, BDE-47 developmental toxicity in porcine embryos in vitro with an IC_50_ of 339.13 μM was studied. Since this is the first time that BDE-47 has been used in early porcine embryos, we referred to a paper on the inhibition of BDE-47 on embryonic stem cell development in mice when determining the initial BDE-47 concentrations. That paper reported that a 100 μM BDE-47 treatment significantly increased the apoptosis rate of mouse embryonic stem cells and significantly decreased the expression of pluripotency genes [33]. Thus, we designed a concentration gradient of 50, 100, and 200 μM. We found that the 50 μM BDE-47 treatment inhibited blastocyst formation insignificantly, but the 100 and 200 μM BDE-47 treatments significantly reduced the blastocyst rate. Therefore, we finally selected 100 μM BDE-47 for the follow-up experiments.

Previous investigations reported that BDE-47 could induce reproductive toxicity, including testicular inflammation and testis damage in mice [34], and increase the serum triiodothyronine levels to impair sperm quality in rats [35]. The present study contributes to the existing literature by investigating the toxicity of BDE-47 on early porcine embryo development in vitro. In this study, we used the four-cell stage and blastocyst stage embryos. The four-cell stage is vital for the development of early porcine embryos because early porcine embryos undergo the zygotic genome activation (ZGA) at the four-cell stage. The ZGA process requires mitochondria to provide enough energy to support the activation of numerous zygotic genomes. Mitochondrial malfunction disrupts the cellular oxidative balance, leading to excessive ROS accumulation. Hence, to analyze oxidative stress and mitochondrial biogenesis, we chose four-cell embryos. We also used blastocysts because the quality and rate of blastocyst production are crucial for assessing early embryonic development.

In this research, Parthenogenetic embryos were employed. Because parthenogenesis technology is mature, the embryo development state is good, a lot of embryos with diploid features are easy to obtain, and there is no bias of paternal contribution; porcine parthenogenetic embryos were used as an excellent model to detect early embryo development [36,37]. We found that exposure to 100 μM BDE-47 significantly decreased the blastocyst rate. Blastocyst formation is associated with cell proliferation, and previous studies have reported that BDE-47 can inhibit proliferation as well as increase apoptosis and autophagy in mouse placental cells, thereby damaging the mouse placenta [26]. Therefore, we further investigated the effects of BDE-47 on embryo pluripotency and autophagy in porcine embryonic cells. The results showed that the pluripotency-related genes *NANOG*, *ESRRB*, and *OCT4* saw a significant decrease in their mRNA levels after exposure to BDE-47. In addition, BDE-47 treatment significantly increased the levels of autophagy-related gene mRNA and the autophagy marker LC3B. Our results suggest that BDE-47 inhibits embryo pluripotency, promotes autophagy, and impairs early development in porcine embryos. These findings are consistent with previous reports.

ROS-mediated oxidative damage to cellular biological molecules, such as DNA, lipids, and proteins, ultimately results in impaired cell and organ functions [38,39,40]. Early embryonic development is also susceptible to oxidative damage [41]. In the present study, IVC medium supplemented with 100 μM BDE-47 markedly increased the level of ROS and decreased the level of GSH. In addition, BDE-47 treatment downregulated the mRNA amounts of the genes *SIRT1*, *SOD1*, *SOD2*, and *GPX,* which were involved in antioxidants. According to our results, BDE-47 induces early embryonic oxidative stress, which is consistent with other research, confirming that BDE-47 induces oxidative stress and apoptosis [42,43].

Mitochondria are important organelles that support cell survival. They produce ATP through oxidative phosphorylation, which provides energy for various cellular activities [44]. Mitochondrial dysfunction can impair germ cell quality, fertilization, and early embryonic development [45]. In the present research, exposure to BDE-47 dramatically reduced the mitochondrial abundance and MMP levels in embryos. The MMP reflects the energy production process of mitochondrial oxidative phosphorylation and is essential for the maintenance of mitochondrial viability [46]. In addition, BDE-47 significantly downregulated the mRNA amounts of the genes *TFAM*, *NRF1*, and *NRF2* that participated in mitochondrial biogenesis. Our study suggests that BDE-47 impairs mitochondrial function, which is consistent with other research [47,48].

As mentioned above, the ER and mitochondria are structurally and functionally related and interact to co-regulate cellular metabolism and homeostasis [49,50]. ERS and mitochondrial homeostasis influence the development and quality of oocytes and early embryos [51,52,53]. In the present study, BDE-47 treatment decreased the ER abundance, increased the amounts of GRP78, an ERS marker, and increased the transcription levels of the ERS-related genes *ATF4*, *uXBP1*, *sXBP1*, *GRP78*, and *CHOP*. Treatment with the ERS inhibitor, PBA, restored these changes and alleviated the damage to the ER caused by BDE-47. Of these, the mRNA upregulation of *GRP78* and *CHOP* did not differ significantly, but in our experiments, the mRNA levels of *GRP78* and *CHOP* genes were higher in BDE-47-exposed embryos than in controls. These results indicate that the mRNA levels of these two genes tend to be upregulated after exposure to BDE-47. The results of immunofluorescence staining also showed that GRP78 was upregulated. In addition, BDE-47 reduced the blastocyst rate and total cell number, while PBA treatment reversed these negative effects. Thus, our study suggests that BDE-47 exposure impairs ER function and embryonic development and that PBA can mitigate this impairment. The results are consistent with other studies [54,55,56]. 

## 5. Conclusions

Taken together, this study suggests that BDE-47 can inhibit the early porcine embryos’ in vitro development through mitochondrial dysfunction and ERS-induced oxidative stress and autophagy.

## Figures and Tables

**Table 1:** Sequences of primers used in RT-qPCR.

Gene	Primer Sequences (5′–3′)	Product Length (bp)
*SOD2*	F: AATCTGAGCCCTAACGGTGG	111
	R: GACGGATACAGCGGTCAACT	
*BECLIN*	F: AGGAGCTGCCGTTGTACTGTTCT	94
	R: TGCTGCACACAGTCCAGGAA	
*GAPDH*	F: TTCCACGGCACAGTCAAG	117
	R: ATACTCAGCACCAGCATCG	
*LC3*	F: TTCAAACAGCGCCGAACCTT	72
	R: TTTGGTAGGATGCTGCTCTCG	
*ATG5*	F: TTGCAGTGGCTGAGTGAACA	78
	R: TCAATCTGTTGGTTGCGGGA	
*OCT4*	F: CCTATGACTTCTGCGGAGGGA	224
	R: TTTGATGTCCTGGGACTCCTCG	
*NANOG*	F: AAGTACCTCAGCCTCCAGCA	235
	R: GTGCTGAGCCCTTCTGAATC	
*SOD1*	F: GTTGGAGACCTGGGCAATGT	104
	R: CGGCCAATGATGGAATGGTC	
*GRP78*	F: GCTCTACTCGCATCCCCAAAG	122
	R: TACACCAGCCTGAACAGCAG	
*SIRT1*	F: ACAGGTTGCAGGAATCCAGAG	137
	R: TAGGACATCGAGGAACCACCT	
*CHOP*	F: CCCCTGGAAATGAGGAGGAG	109
	R: CTCTGGGAGGTGTGTGTGAC	
*ESRRB*	F: CCGGACAAACTCTACGCCAT	134
	R: GAGAAGCCTGGGATGTGCTT	
*ATF4*	F: AGTCCTTTTCTGCGAGTGGG	80
	R: CTGCTGCCTCTAATACGCCA	
*GPX*	F: GGTCTCCAGTGTGTCGCAAT	106
	R: TCGATGGTCAGAAAGCGACG	
*uXBP1*	F: CATGGATTCTGACGGTGTTG	106
	R: GTCTGGGGAAGGACATCTGA	
*TFAM*	F: CGCTCTCCGTTCAGTTTTGC	138
	R: TGCATCTGGGTTCTGAGCTTT	
*sXBP1*	F: GGAGTTAAGACAGCGCTTGG	142
	R: GAGATGTTCTGGAGGGGTGA	
*NRF1*	F: CCTGTGAGCATGTACCAGACT	148
	R: ACTGTTCCAACGTCACCACCT	
*p62*	F: AAGAACGTAGGGGAGAGTGTG	80
	R: TTCCCTCCATGTTCCACGTC	
*NRF2*	F: AGCGGATTGCTCGTAGACAG	110
	R: TTCAGTCGCTTCACGTCGG	

F, Forward primer; R, Reverse Primer.

## Data Availability

Data are contained within the article.
